# Systemic effect of mineral aggregate-based cements: histopathological analysis in rats

**DOI:** 10.1590/1678-7757-2016-0634

**Published:** 2017

**Authors:** Lucas da Fonseca Roberti Garcia, Claudia Huck, Fernando Augusto Cintra Magalhães, Pedro Paulo Chaves de Souza, Carlos Alberto de Souza Costa

**Affiliations:** 1 Universidade Federal de Santa Catarina, Centro de Ciências da Saúde, Departamento de Odontologia, Área de Endodontia, Florianópolis, SC, Brasil.; 2 Univ. Estadual Paulista, Faculdade de Odontologia de Araraquara, Departamento de Odontologia Restauradora, Araraquara, SP, Brasil.; 3 Univ. Estadual Paulista, Faculdade de Odontologia de Araraquara, Departamento de Fisiologia e Patologia, Araraquara, SP, Brasil.

**Keywords:** Endodontics, Biocompatible materials, Inflammation, Liver, Kidney

## Abstract

**Objective:**

Several studies reported the local tissue reaction caused by mineral aggregate-based cements. However, few studies have investigated the systemic effects promoted by these cements on liver and kidney when directly applied to connective tissue. The purpose of this *in vivo* study was to investigate the systemic effect of mineral aggregate-based cements on the livers and kidneys of rats.

**Material and Methods:**

Samples of Mineral Trioxide Aggregate (MTA) and a calcium aluminate-based cement (EndoBinder) containing different radiopacifiers were implanted into the dorsum of 40 rats. After 7 and 30 d, samples of subcutaneous, liver and kidney tissues were submitted to histopathological analysis. A score (0-3) was used to grade the inflammatory reaction. Blood samples were collected to evaluate changes in hepatic and renal functions of animals.

**Results:**

The moderate inflammatory reaction (2) observed for 7 d in the subcutaneous tissue decreased with time for all cements. The thickness of inflammatory capsules also presented a significant decrease with time (P<.05). Systemically, all cements caused adverse inflammatory reactions in the liver and kidney, being more evident for MTA, persisting until the end of the analysis. Liver functions increased significantly for MTA during 30 d (P<.05).

**Conclusion:**

The different cements induced to a locally limited inflammatory reaction. However, from the systemic point of view, the cements promoted significant inflammatory reactions in the liver and kidney. For MTA, the reactions were more accentuated.

## Introduction

Mineral Trioxide Aggregate (MTA) is one of the most used biomaterials in endodontics currently^3^. It is a silicate-based cement, basically composed of purified Portland cement (75.0%) (wt) added to dehydrated CaSO_4_ (5.0%) and Bi_2_O_3_ (20.0%), the latter being responsible for its radiopacity^2^. Because of its hydraulic nature, after the addition of water, the setting process of the cement begins by initially forming a hydrated silica gel^3^.

There is few information regarding the MTA cement manufacturing process. However, the companies responsible for this procedure affirm that they produce the cement under conditions that guarantee its safe use. Portland cement, the main raw material of MTA, has several compounds in its formulation, the following being the mostly used ones: SiO_2_ (21.2%); CaO (68.1%); Al_2_O_3_ (4.7%); MgO (0.48%) and Fe_2_O_3_ (1.89%)^3^. Despite Portland undergoing a purification process before being used, studies have proved the presence of heavy metals in the composition of MTA, among them As, Cr and Pb^11^ ^,^ ^12^. Nevertheless, it is possible to find these metals at levels below the safe limits proposed by international specifications^12^.

In spite of its proven biocompatibility and bioactivity^20^ ^,^ ^21^, undesirable effects of Bi_2_O_3_ have been pointed out on the rate of Ca++ ions released from MTA, thus compromising its performance as a reparative material^3^ ^,^ ^25^. Furthermore, studies have reported that heavy metal salts, mainly Bi and Cd, are responsible for the expression of the heme oxygenase-1 enzyme (HO-1) in different cell lineages, compromising their regulatory functions and protective mechanisms^4^ ^,^ ^22^.

In a recent study, Khalil and Eid^18^ (2013) reported adverse inflammatory reactions caused by MTA in the liver and kidneys of rats, questioning the systemic compatibility of this type of cement. Therefore, new biomaterials have been developed to minimize adverse reactions caused by reparative cements when in contact with living tissues^13^.

A novel calcium aluminate-based cement (EndoBinder - Patent Number PI0704502-6) with similar clinical applications of MTA was developed at the Federal University of São Carlos (UFSCar - Brazil) to address the deficiencies of the silicate-based cement^3^ ^,^ ^11^ ^,^ ^12^ ^,^ ^25^. Similarly, different radiopacifiers, such as ZnO, have been proposed as a feasible alternative to the compounds currently used for this purpose^26^.

Therefore, the aim of this *in vivo* study was to investigate the local (subcutaneous) and systemic (liver and kidney) effects of MTA and a new reparative calcium aluminate-based cement (EndoBinder) containing different radiopacifiers in rats. The null hypothesis tested was that there would be no significant difference in the inflammatory response of cements in the different evaluated tissues.

## Material and methods

### Animals

The entire study was developed according to the guidelines of the Research Ethics Committee on the Use of Animals (Process CEUA No. 3/2013), and the National Institutes of Health guide for the care and use of laboratory animals (NIH Publications No. 8023, revised 1978). For this study, 40 male rats (*Rattus novergicus,* Wistar), weighing 300 g, were selected. The number of specimens *per* group was determined based on other biological studies that used a similar quantity of animals^1^ ^,^ ^13^ ^,^ ^14^. In addition, sample size was calculated to set a number of needed specimens to detect effective statistical difference of 5% (a = .05) among control and experimental groups. Animals were kept in plastic cages (40×32×17 cm) especial for this purpose, accommodated in an acclimatized bioterium (temperature: 21-23°C/relative humidity: 60±5%/12 h light-dark cycle), and received balanced rations (Nuvilab, Colombo, PR, Brazil) and water *ad libitum* during the experiment.

### Experimental design and mineral aggregate-based cements treatment

The tested cements were distributed as it follows: EndoBinder (Binderware, São Carlos, SP, Brazil) + 20% (wt) Bi_2_O_3_ (EBBO Group), EndoBinder + 20% ZnO (EBZnO Group), and White MTA (Ângelus, Londrina, PR, Brazil) (WMTA Group). Then, they were manipulated according to the manufacturers’ recommendations. For EndoBinder, the proportion of 1 g of powder to 0.21 mL of distilled water was used, whereas for White MTA, the recommended proportion was of one dose of powder (0.15 mg) to 1 drop (0.5 mL) of distilled water.

After manipulating the cements, pre-sterilized polyethylene tubes measuring 1.5 mm of internal diameter and 10 mm of length were filled with 0.10 g of cements using a sterile Lentulo spiral (Dentsply/Maillefer, Ballaigues, Switzerland)^13^. Before filling the tubes with the tested cements, one of their extremities was heat-sealed to avoid cement extravasation^13^.

The animals were anesthetized by intraperitoneal administration with a 10% solution of ketamine chloride (Ketamina Agener, União Química Farmacêutica Nacional S/A, Embu-Guaçu, SP, Brazil) (75 mg/kg) and xylazine (Dopazer, Laboratórios Calier S.A, Barcelona, Spain) (10 mg/kg). After trichotomy of the animal's dorsum, to perform the surgical procedure, the area was disinfected with a 5% Povidone-iodone (PVP-I) solution. A 5 mm-long incision was made at the center of the animal's dorsum with a No.15 scalpel blade. The subcutaneous tissue was laterally divulsed with a blunt-tipped scissor, from the center of the incision. A surgical pocket with an average depth of 20 mm was obtained, in which the polyethylene tube with the cement under test was implanted longitudinally, in parallel to the incision, letting the open extremity in direct contact with the subcutaneous tissue. Each cement was implanted in groups of 10 animals. Each group of 10 animals was distributed into two sub-groups (n=5) according to the time interval of analysis (7 and 30 d). In other 10 animals, surgical pockets were prepared, in which empty polyethylene tubes were implanted, thus establishing the negative control group. After concluding the polyethylene tubes implants, the wound was sutured with a 3/0 silk thread (Ethicon, São José dos Campos, SP, Brazil).

After experimental time intervals of 7 and 30 d, animals were anesthetized for blood sample collection (n=5). Blood (5 mL) was collected under vacuum by saphenous vein puncture^18^ of the retro-orbital plexus with the aid of heparinized capillary tubes (BD Vacultainer, BD - Benton, Dickinson and Company, Franklin Lakes, NJ, USA). Afterwards, the animals were sacrificed by anesthetic overdose to perform biopsies of subcutaneous tissues, livers and kidneys. To standardize the sampling, only the left kidney of each animal was collected for analysis. After biopsies, the tissues and organs collected were immediately immersed in a 10% formalin solution (Merck, Darmstadt, Germany), where they remained for 24 hours at room temperature. After this period, all surgical parts were submitted to routine laboratory processing. Polyethylene tubes were removed before tissue sectioning, then the tissues were sectioned longitudinally, in 5 μm-thick semi-serial cuts, considering 10 cuts were discarded and 5 were stained with hematoxylin-eosin (Merck).

### Histopathological examination

Histopathological examination was performed blindly and double-checked by two previously calibrated examiners with a concordance index of 94% between them, using an optical light microscope (Axio Star Plus, Carl Zeiss, Oberköchen, Germany). The local (subcutaneous tissue) and systemic (liver and kidney) biocompatibility of the cements were evaluated considering the presence of the following histopathological events: inflammatory infiltrate (polymorphonuclear and mononuclear cells), fibroangioblastic proliferation and vascular congestion (fibroblasts and blood vessels) and macrophagic activity (mononuclear phagocytes and multinucleated giant cells)^5^ ^,^ ^13^. In addition to these tissue events, the presence of micro and macrovesicular steatosis, and the occurrence of apoptosis in hepatic cells were also considered to grade the inflammatory reaction in the liver^5^, whereas, for the kidney, the presence of hypercellularity in the cortex was used^18^. According to the ISO 7405:2008 specification^17^ (2008), and based on the tissue responses stimulated by different cements and the control group, a score was used to quantify the extension of these events, with tissue inflammation being classified as it follows: (0) absent, (1) discrete, (2) moderate, and (3) severe. In the case of subcutaneous implants, the thickness (μm) of the inflammatory capsule was also measured using the Axio Vision 4.6 software (Axio Star Plus).

### Blood sample analyses

The blood samples previously collected in heparinized tubes were transferred to Eppendorf tubes (Eppendorf do Brasil Ltda., São Paulo, SP, Brazil), and then centrifuged to separate the plasma and the solid constituents of the blood. After centrifugation, 1 mL of plasma was collected and submitted to biochemical analysis to quantify alanine aminotransferase (ALT) and aspartate aminotransferase (AST) serum enzymes, which are indicators of liver functions, and urea and creatinine levels (of renal function).

### Statistical analysis

After testing the normality of the sample (Shapiro-Wilk test - P>.05), values obtained in different experiments were statistically compared to the two-way analysis of variance (ANOVA) and the Bonferroni's significant difference *post hoc* test (P<.05). Statistical analysis was performed with the OriginPro 8 SRO program (OriginLab Corp., Northampton, MA, USA).

## Results

### Subcutaneous tissue

Frequencies observed for each of the histopathological events evaluated in different experiment time intervals can be seen in Table 1.

**Table 1:** Frequencies of histopathological events observed in the subcutaneous tissue in each group at different experimental periods

Histopathological events	Experimental periods							
	7 Days				30 Days			
	EBBO	EBZnO	WMTA	*C	EBBO	EBZnO	WMTA	*C
Inflammatory infiltrate (polymorphonuclear and mononuclear)	2	2	2	1	1	1	1	1
Fibroblasts	2	2	2	1	2	2	2	1
Blood vessels	2	2	2	1	1	1	2	1
Macrophages	2	2	2	1	1	1	1	1
Giant multinuclear cells	2	2	2	1	0	0	1	0
Inflammatory index	2	2	2	1	1	1	1	1

* C=Negative control group.

Score: (0) absent; (1) discrete; (2) moderate; (3) severe. n=5

### EBBO group

**Figure 1:**
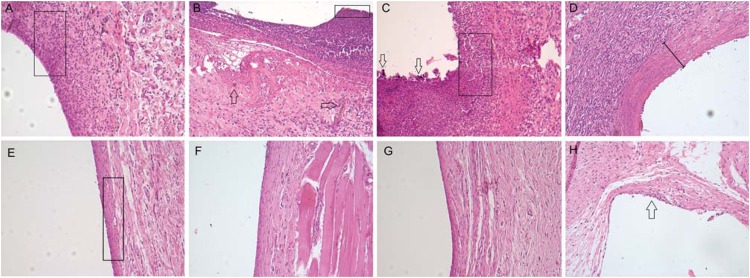
Photomicrographs of histological sections (5 μm) of subcutaneous tissue stained with hematoxylin & eosin. (A) EBBO 7 d: We observe moderate inflammatory infiltrate with predominance of polymorphous and mononuclear cells (box) at the tube opening where the cement was present. Original magnification x125; (B) EBZnO 7 d: In addition to moderate local inflammatory infiltrate, it was possible to observe a thin layer of tissue necrosis in contact (box), and material residues dispersed within the tissue (arrows). Original magnification x125; (C) WMTA 7 d: We noted the layer of necrosis at the tube opening; and the presence of dispersed cement fragments (arrows) with moderate and predominantly mononuclear inflammatory infiltrate (box). This reactionary pattern of the tissue was similar for all the tested versions of EndoBinder. Original magnification x125; (D) Control 7 d: Details of the inflammatory capsule formed at the tube opening (bar), where it was possible to observe discrete inflammatory infiltrate. Original magnification x125. (E) EBBO, (F) EBZnO, and (G) WMTA 30 d: We were able to observe a thin, whole and continuous fibrous capsule, containing only few mononuclear inflammatory cells at the tube opening (box), demonstrating that the tissue exposed to the different versions of EndoBinder and MTA cement could repair itself with time. Original magnification x125. (H) Control 30 d: Discrete extent of inflammatory infiltrate with few polymorphous and multinuclear cells (arrow) without macrophagic activity. Original magnification x125.

In the initial time interval of 7 d, we observed an ample, loose and disorganized inflammatory capsule adjacently to the tube opening where the material was placed in contact with the tissue. This capsule had moderate inflammatory infiltrate mainly characterized by the presence of polymorphous and mononuclear cells. We noted discrete collagenization associated with fibroangioblastic proliferation in most samples. We also observed giant multinuclear cells and macrophages phagocyting biomaterial residues dispersed at the tube opening. Small areas of tissue necrosis could be present within the inflammatory capsule. After 30 d, we observed a significant reduction in the extent of inflammatory infiltrate with discrete local macrophagic activity. The inflammatory capsule at the tube opening has shown to be fibrous, well organized and associated with discrete local inflammatory infiltrate, a characteristic of the tissue repair process (Figures 1A and E). In the time interval of 30 d, we considered the general inflammatory index of 1 (discrete).

### EBZnO group

As described in the previous group, in the initial time interval (7 d), it was possible to observe the formation of an ample inflammatory capsule with moderate inflammatory infiltrate at the tube opening, the main area of analysis. We also noted fibroangioblastic proliferation associated with the presence of macrophages phagocyting dispersed biomaterial residues and mononucleated giant cells in the region. In addition, we noted an area of necrosis similar to the EBBO Group in this initial time interval of analysis. After 30 d, there was a reduction in inflammatory infiltrate with discrete macrophagic activity and a significant reduction in the quantity of blood vessels in the tissue. No areas of mineralization and tissue necrosis occurred at the final period of analysis (Figures 1B and F). As observed in EBBO Group, after a period of 30 d, we established a general inflammatory index of 1 (discrete) for the EBZnO Group.

### WMTA group

After 7 d, at the tube opening, it was possible to identify the formation of a disorganized capsule with moderate fibroangioblastic proliferation and numerous blood vessels associated with moderate inflammatory infiltrate. We found macrophages and giant multinucleated cells phagocyting dispersed material residues, as well as areas of tissue necrosis and absence of locally mineralized *foci.* At the final period (30 d), we observed a significant decrease in the extent of inflammatory infiltrate with discrete macrophagic activity. We identified no signs of necrosis at the end of analysis, but there was the formation of a thin fibrous capsule at the tube opening, as observed in a normal repaired tissue (Figures 1C and G). The general inflammatory index for WMTA in this time interval of 30 d was also 1 (discrete).

### Control group

In the time interval of 7 d, there was a formation of a thin inflammatory capsule with a discrete quantity of polymorphous and mononuclear. Discrete fibroangioblastic proliferation and local collagenization occurred with neoformation of blood vessels and without signs of congestion. A discrete quantity of mononuclear phagocytes was noted without signs of tissue necrosis. After 30 d, we detected a significant decrease in inflammatory infiltrate and local macrophagic activity, but we did not see giant multinuclear cells at the site. We observed a fibrous capsule with many fibroblasts at the middle of few small caliber blood vessels, which characterizes complete tissue repair (Figures 1D and H). For the Control Group, the general inflammatory index was 1 (discrete) at 30 d.

### Inflammatory capsule thickness

**Figure 2:**
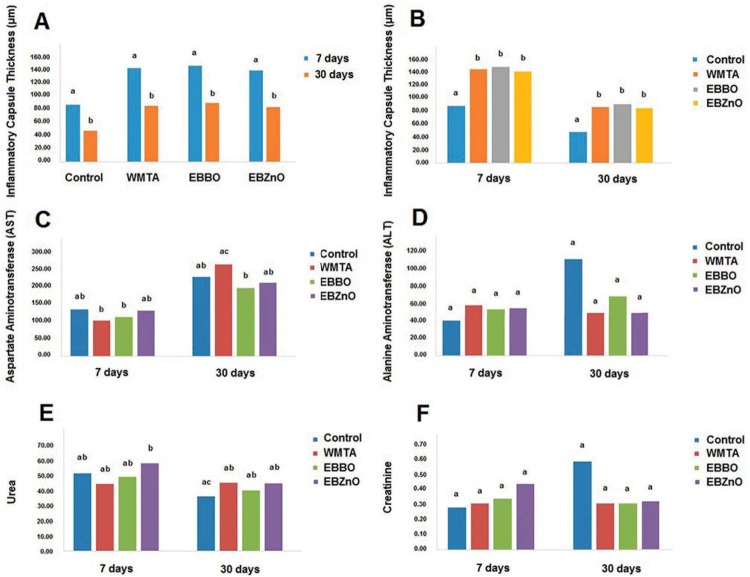
(A and B) Graphic representation of mean values (μm) of inflammatory capsule thickness at different experimental periods; (C) Graphic representation of aspartate aminotransferase (AST) levels according to experimental periods; (D) Graphic representation of serum alanine aminotransferase (ALT) levels according to experimental periods; (E) Graphic representation of urea levels according to experimental periods; (F) Graphic representation of creatinine levels according to experimental periods. Different lower case letters over bars indicate statistically significant difference (2-way ANOVA, Bonferroni test, P<.05). n=5

The mean thickness values of the inflammatory capsule of different groups with distinct experimental times can be seen in Figures 2A and B.

During time of analysis (7 and 30 d), we verified the formation of an inflammatory capsule of significantly decreasing extent for all groups (P<.05). The capsule formed adjacently to the different tested cements in the period of 7 d was significantly thicker compared to the Control Group (P<.05). We observed this trend at the end of analysis Thickness values were as it follows: EBBO (86.09 μm); EBZnO (82.80 μm); and WMTA (85.43 μm), with no statistical difference among them (P>.05). However, these groups were statistically different from the Control Group (46.75 μm) (P<.05).

### Liver and kidney

The frequencies observed for each of the histopathological events evaluated in the different experimental time intervals can be seen in Table 2.

**Table 2:** Frequencies of histopathological events observed in the liver and kidney in each group at different experimental periods

Histopathological events	Liver								Kidney							
	Experimental periods															
	7 Days				30 Days				7 Days				30 Days			
	EBBO	EBZnO	WMTA	*C	EBBO	EBZnO	WMTA	*C	EBBO	EBZnO	WMTA	*C	EBBO	EBZnO	WMTA	*C
Inflammatory infiltrate (polymorphonuclear and mononuclear)	2	2	2	0	2	2	2	0	2	2	2	0	1	1	2	0
Fibroblasts	2	2	2	1	1	1	2	0	2	2	2	1	2	2	2	0
Blood vessels	3	3	3	1	2	2	2	0	2	2	2	1	1	1	2	1
Macrophages	2	2	2	0	1	1	2	0	1	1	1	0	1	1	1	0
Giant multinuclear cells	1	1	2	0	1	1	1	0	1	1	1	0	1	1	1	0
Steatosis	2	2	2	0	1	1	2	0	–	–	–	–	–	–	–	–
Apoptosis	1	1	2	0	1	1	1	0	–	–	–	–	–	–	–	–
Hipercellularity	–	–	–	–	–	–	–	–	2	2	2	0	1	1	2	0
Inflammatory index	2	2	2	0	1	1	2	0	2	2	2	0	1	1	2	0

* C=Negative control group.

Score: (0) absent; (1) discret; (2) moderate; (3) severe. n=5

### EBBO group

**Figure 3:**
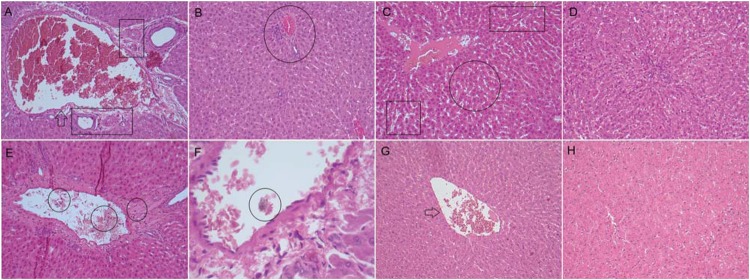
Photomicrographs of histological sections (5 μm) of liver stained with hematoxylin and eosin. (A) EBBO 7 d: Details of tributary vessel of distorted, dilated and congested hepatic portal system with ruptured endothelial layer (arrow). The stroma presents moderate inflammatory infiltrate dispersed in the surrounding parenchyma, as well as areas of microvesicular steatosis. Note the intense angioblastic proliferation determining the formation of numerous capillaries at the site of analysis (box). Original magnification x125; (B) EBBO 30 d: In general, we observed a tissue with normal characteristics despite the presence of persistent discrete inflammatory infiltrate associated with a few congested blood vessels (circle). Original magnification x125; (C) EBZnO 7 d: We also observed dilated and congested blood vessels, and moderate inflammatory infiltrate with predominance of polymorphous and mononuclear cells. Note the granular aspect and the increased size of the hepatocytes, characterizing a cell degeneration process (circle). We could also detect several intracellular edema areas (box). Original magnification x125; (D) EBZnO 30 d: Histological section showing tissue with discrete mononuclear cells infiltration and residual areas of edema. Original magnification x125; (E) WMTA 7 d: Inflammatory infiltrate of greater extent compared to the groups treated with EndoBinder. Note the presence of polymorphous and mononuclear cells within and surrounding the blood vessels (circle). Original magnification x125; (F) WMTA 30 d: Details of a tributary blood vessel of the hepatic portal system. Note the presence of a foreign body within the vessel, which suggests the presence of dispersed residual material (circle). Original magnification x320; (G) Control 7 d: Tributary vessel of the hepatic portal system with normal morphology and a few signs of congestion and intact endothelial layer (arrow). Original magnification x125; (H) Control 30 d: Details of a normal tissue obtained from a healthy animal liver. Original magnification x125

**Figure 4:**
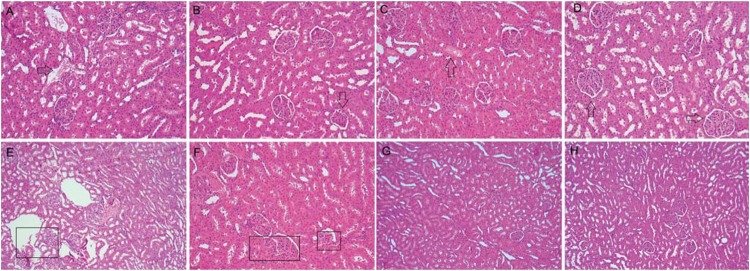
Photomicrographs of histological sections (5 μm) of kidney stained with hematoxylin and eosin. (A) EBBO 7 d: We observed renal corpuscles containing preserved glomeruli, as well as intact proximal and distal tubule. However, note dilated and congested blood vessels associated with discrete local inflammatory infiltrate (arrow). Original magnification x125; (B) EBBO 30 d: The histological section shows a tissue with normal characteristics, although it has been noted, in a renal corpuscle,a rupture of the visceral leaflet, and a distortion of capsular space (arrow). Original magnification x125; (C) EBZnO 7 d: It was possible to observe the presence of diffuse areas of hypercellularity and congested blood vessels (arrow) associated with the formation of numerous capillaries among the few mononuclear inflammatory cells. Note that several renal corpuscles present loss of capsular space and rupture of the visceral and parietal leaflets. Original magnification x125; (D) EBZnO 30 d: As observed for the EBBO Group at the same period (30 d), the tissue shows normal histological characteristics with renal corpuscles and other well-preserved renal structures (arrow). Original magnification x125; (E) WMTA 7 d: The histological section shows several dilated blood vessels and endothelial disruption (box), characterizing areas of hemorrhage and wide edema formation. Note hydropic degeneration of cells that comprise the distal and proximal tubules, which appear distorted and disorganized. It is also possible to detect many renal corpuscles with ruptured visceral and parietal leaflets, and inflammatory cells in the glomeruli. Original magnification x125; (F) WMTA 30 d: Despite the renal corpuscles, a better organized structure (box), it is possible to observe the persistence of numerous congested blood vessels. Note that the proximal and distal tubules are better constituted and distributed. These features demonstrate that the kidney is in continuous process of tissue repair. Original magnification x125; (G and H) Control 7 and 30 d: The histological section shows a tissue with normal histological characteristics. It is possible to observe several renal corpuscles with organized glomerulus, as well as intact parietal and visceral leaflets preserving the capsular space. Note the presence of distal and proximal renal tubules with normal characteristics. Original magnification x125

At the time interval of 7 d, we observed inflammatory infiltrate of moderate extent in the liver of the animals close to the blood vessels, and mononuclear cells inside the tributary vessels of the hepatic portal system. Most of these vessels seemed distorted, dilated and had a ruptured endothelial layer. In addition, we noted the congestion of these vessels and the presence of microvesicular steatosis, characteristic of a degenerative process. After 30 d, there was still persistent inflammatory infiltrate, however, with notable reduction in mononuclear phagocytes. It was also possible to observe hepatocytes in a process of apoptosis; in a smaller quantity, however (Figures 3A and B). At 7 d, in the kidney, the main observed events were vascular congestion and moderate increase of local cellularity, triggering an increase in renal cortex density, typical of a tissue degenerative process. However, at the period of 30 d, we noted a reduction in the extent of these events in the affected areas (Figures 4A and B). At the end of the analysis, we considered 1 (discrete) as the general inflammatory index for the liver and kidney.

### EBZnO group

The histopathological events observed in both time intervals of analysis were similar to those from the previous groups, both for livers and kidneys. We were able to observe an inflammatory infiltrate of polymorphous and mononuclear cells of moderate extent in the initial period. Most blood vessels seemed congested, and had a ruptured endothelial layer, which leads to a process of flbroangioblastic proliferation, with the formation of various accessory vessels. In addition, we noted the presence of moderate microvesicular steatosis and several areas of intracellular edema. At the period of 30 d, we also noted a small quantity of hepatocytes in mitosis and apoptosis and a decrease in inflammatory infiltrate (Figures 3C and D). In the kidney, we observed vascular congestion and hypercellularity with an increase in the renal cortex density, followed by reduction of these features at the final period (30 d) (Figures 4C and D). After 30 d, the general inflammatory index for both organs was also 1 (discrete).

### WMTA group

At the period of 7 d, despite involving larger areas than the ones observed in the groups treated with different versions of EndoBinder, we noted the presence of an inflammatory infiltrate of moderate extent in the liver. Blood vessels seemed congested with inflammatory polymorphous and mononuclear cells within and adjacently to them. In addition, we observed microvesicular steatosis and several areas of regional necrosis. In only one of the samples, we detected a foreign body in one of the tributary vessels of the hepatic portal system, associated with the dispersed residual material tested in this group. After 30 d, the inflammatory process remained persistent and associated with moderate fibroangioblastic proliferation, with several hepatocytes that seemed to be undergoing mitosis and apoptosis. We also detected punctual areas of necrosis and hydropic degeneration in the tissue (Figures 3E and F). After 7 d, we observed vascular congestion and hypercellularity in the kidney, with an increase in renal cortex density and the presence of malformed glomerulus. In some samples, it was possible to detect residues of degenerated glomerulus. After 30 d, we observed the intensity of diminished renal response with tissue reorganization, a characteristic of a repair process. However, this decrease in the extent of the observed histological events was lower than that observed in the groups treated with EndoBinder (Figures 4E and F). After 30 d, we considered 2 (moderate) as the general inflammatory index for the liver and kidney.

### Control group

In the control group, animals underwent the same operative procedures, but we did not expose them to mineral aggregate-based cements. We observed tributary vessels of the hepatic portal system with normal morphology in the liver. In the kidney, the renal cortex outline exhibited no signs of thickening or rupture. Histological sections of the liver showed no hepatic microvesicular steatosis in any of the time intervals of analysis and no sign of necrosis. We detected discrete inflammatory infiltrate after 7 d, which was not present at the period of 30 d (Figures 3G and H, and Figures 4G and H). The general inflammatory index for both organs was of 0 (absent) at the final period of analysis.

### Blood sample analyses

The mean values obtained for ALT, AST, urea, and creatinine levels can be seen in Figure 2C-F.

Only WMTA showed significant increase in the AST levels between time intervals of 7 and 30 d (P<.05). AST levels for WMTA were statistically different compared to the EBBO Group in both time intervals. For the ALT levels, there were no significant difference between the studied groups (P>.05). Whereas for urea, only the EBZnO Group (7 d) showed significant difference compared to the Control Group (30 d) (P<.05). For creatinine, there were no differences between the studied groups and time intervals of analysis (P>.05).

## Discussion

This study aimed to investigate the local and systemic effect of different mineral-aggregate-based cements (MTA and EndoBinder - containing different radiopacifiers). Based on the results obtained, we were able to state that we partially accepted the tested hypothesis as all cements caused a similar inflammatory response when locally implanted into subcutaneous tissue. However, the systemic results were adverse when we analyzed the action of these materials on the liver and kidney.

Diverse previous studies have evaluated the tissue reactions caused by calcium silicate-based cements, such as MTA, and new calcium aluminate-based cements, such as EndoBinder^1^ ^,^ ^13^ ^,^ ^24^. However, few studies have evaluated the systemic effect of these cements when applied directly with connective tissue^9^ ^,^ ^10^ ^,^ ^18^. The ISO 10993-1 specification^15^ (2006) determines that systemic toxicity of all biomaterials that contacted blood, such as those evaluated in this study, must be assessed. According to Culliton, et al.^6^ (1981), the subcutaneous implantation protocol is a reliable method for determining the systemic toxic effects of biomaterials.

Despite the reliability of the systemic toxicity tests that use animal models, the findings observed in this study should not be considered for humans^18^. However, these preliminary results are important and must be considered regarding the clinical use of the tested materials.

Bismuth oxide (Bi_2_O_3_), a compound used as a radiopacifier of MTA, has been questioned because of its deleterious effects on important biological properties of the cement, such as its reparative capacity^7^. Bi_2_O_3_ is known for interfering directly in the hydration mechanism of MTA^3^, reducing the release of Ca++ ions^25^. This fact appears to harm the good biologic performance of MTA as direct capping agent, because it inhibits the synthesis and deposition of reparative hard tissue on exposed pulps, and the consequent formation of mineral barrier^3^ ^,^ ^25^.

For this reason, in this study we evaluated different versions of EndoBinder containing two distinct types of radiopacifiers. ZnO has been outstanding as an alternative radiopacifier for prosthetic materials and dental implants because of its adequate biological compatibility and because it does not promote significant changes in the chemical and physicomechanical properties of these materials^26^. In addition to the version containing 20% of ZnO in its composition, we tested a version of EndoBinder containing 20% of Bi_2_O_3_. Therefore, we were able to answer questions regarding the effect of this compound on the biological compatibility of these cements.

Over a decade ago, Yaltirik, et al.^30^ (2004) reported a chronic inflammatory process promoted by MTA 60 d after its subcutaneous implantation, in which they could observe macrophages and giant multinucleated around the particles of cement dispersed at the middle of the tissue. On the other hand, in the present study, we characterized the tissue reaction caused by the different materials tested, including MTA, by subcutaneous implantation protocol, by the presence of a discrete inflammatory reaction at the end of 30 d of analysis. These data are by Garcia, et al.^13^ (2014), who showed that both MTA and EndoBinder caused discrete local inflammatory reaction 30 d after implantation, which is associated with the formation of a well-organized capsule, a characteristic of the tissue repair process. At the end of 90 d, the authors observed no sign of inflammation at the tube opening, the main area of analysis. According to the ISO 7405 specification^17^ (2008), a certain material presents acceptable compatibility when it causes moderate inflammation 15 d after subcutaneous implantation, decreasing tissue reaction after 90 d. It is known that all materials applied directly with living tissues cause some type of local irritant action. However, more important than irritation caused by these materials is its persistence^29^. Thus, the results of recent research appear to indicate the local biocompatibility of MTA and EndoBinder cement. Moreover, in this study, this positive biological property of EndoBinder did not depend on the radiopacifier in the cement composition.

Although previous studies have shown that Bi_2_O_3_ diminished the release of Ca^++^ ions from MTA^3^ ^,^ ^25^, good tissue repair observed at the end of 30 d showed that, at least locally, the Ca^++^ ions released by it did not change^28^; this was one of the main reasons for the positive results on tissue biocompatibility obtained in this study. However, we observed significant changes in the liver and kidney of animals when we compared the experimental groups to the Control group, demonstrating that despite the good local repair results, the tested cements presented some systemic toxic potential.

For all the cements, we observed areas of microvesicular steatosis in the liver, a degenerative phenomenon that could lead to macrovesciular steatosis if we did not remove the pathogenic agent^18^ ^,^ ^27^. In the groups treated with the cements, such phenomenon can represent a certain degree of liver toxicity^18^ ^,^ ^27^.

Although all cements caused significant changes in the liver and kidney when compared to the control group, the intensity of the events observed was greater for MTA. The extent of the observed inflammatory infiltrate for MTA did not decrease during the periods of analysis; and the areas of tissue necrosis and hidropic degeneration can explain the slower process of recovery in the liver with the implementation of this cement. The analysis of the blood samples proved these results because the functions of the liver increased significantly for MTA after 30 d, indicating a possible hepatotoxicity^18^ ^,^ ^27^. Histologically, we could observe the same in the kidney; we could still observe residues of degenerated glomerulus at the end of the time interval of 30 d.

Diverse heavy metals have been detected in the composition of MTA, among them arsenic^11^ ^,^ ^12^. However, the quantity released by the cement is many times lower than the value considered safe (2 mg/kg^−1^) by the ISO 9917-1 specification^16^ (2007), which it does not compromise its clinical application^11^ ^,^ ^12^. It has also been demonstrated that EndoBinder releases diverse heavy metals, such as lead, chrome and arsenic^12^. As observed for MTA, the levels found for EndoBinder were lower than those considered safe by the ISO 9917-1 specification^16^ (2007). In the case of arsenic, the dose considered lethal ranges from 2 to 3 mg/Kg of body weight. Thus, 140 to 210 mg of arsenic would be required to poison an individual weighing 70 Kg^23^. Considering that the quantity of MTA used during a clinical procedure is less than 1.0 g, the quantity of arsenic present in the cement is much lower than the lethal dose^23^. However, from the clinical point of view, despite the low levels of arsenic released by the cements^12^, the quantity necessary to cause significant changes in tissues and vital organs that are at a certain distance from the site of application has not yet been elucidated.

Clinical procedures that involve the treatment of exposed living tissues, such as cases of pulpotomy, direct pulp protection, treatment of root perforations and furca, as well as parendodontic surgeries are part of daily routine, and in most cases, require the use of cements such as EndoBinder and MTA^14^. When particles of arsenic come into direct contact with blood, red globules absorb them and take them to the liver and kidneys by the blood stream^19^. We point out in this study that the cements applied in surgical pockets created in the subcutaneous connective tissue of rats, which led to the contact of the tested materials with the animals’ blood. Other *in vivo* studies demonstrated that once pentavalent arsenic is absorbed by the liver, it becomes methylated into its trivalent form, which is less toxic and more easily eliminated^8^ ^,^ ^11^. However, if the liver is not capable of metabolizing the arsenic in a reasonable time, it causes irreparable damage to the organ, even when the doses are not considered lethal^8^ ^,^ ^11^. The results observed in the histopathological analysis of the liver and kidney led us to believe that these heavy metals in the composition of EndoBinder and MTA, particularly arsenic can play a relevant clinical role in the systemic toxicity caused by these cements^8^, and that their action is time-dependent.

In a recent research, Demirkaya, et al.^9^ (2016) have reported that the levels of aluminium in blood plasma and liver were higher in rats with MTA-like cements implanted into their dental extraction sockets, indicating that aluminium could be released from calcium silicate cements into the blood stream. Moreover, in another study, Demirkaya, et al.^10^ (2016) demonstrated that aluminium levels increased in the brains of rats submitted to these cements implantation. According to the authors, heavy metals released from mineral aggregate-based cements can promote a potential neurological damage to brains because of oxidative stress induction by transitional upregulation of antioxidant enzymes. Similarly, it is possible to considered that the presence of aluminium in liver and kidney would lead to changes in ALT, AST, urea, and creatinine levels, in addition to the damage histologically observed in these organs^10^.

## Conclusion

In summary, the different mineral aggregate-based cements investigated in this study promoted limited local inflammatory reactions on subcutaneous tissue, which decreased with time. Thus, it could be stated that all cements were locally biocompatible. Systemically, all cements caused adverse histological reactions on liver and kidney; there were more accentuated reactions for MTA that persisted at the end of 30 d. In addition, it is worth to emphasize that these systemic reactions can not be considered irreversible, and further researches using longer periods of analysis must be conducted.

## References

[1] Aguilar FG, Roberti Garcia LF, Panzeri Pires-de-Souza FC (2012). Biocompatibility of new calcium aluminate cement (EndoBinder). J Endod.

[2] Belío-Reyes IA, Bucio L, Cruz-Chavez E (2009). Phase composition of ProRoot mineral trioxide aggregate by X-ray powder diffraction. J. Endod.

[3] Camilleri J (2008). Characterization of hydration products of mineral trioxide aggregate. Int Endod J.

[4] Cavicchi M, Gibbs L, Whittle BJ (2000). Inhibition of inducible nitric oxide synthase in the human intestinal epithelial cell line, DLD-1, by the inducers of heme oxygenase 1, bismuth salts, heme, and nitric oxide donors. Gut.

[5] Cornianu M, Dema A, Tăban S, Lazăr D, Lazăr E, Costi S (2005). Hepatic steatosis associated with hepatitis C virus infection. Rom J Morphol Embryol.

[6] Culliton CR, Meenaghan MA, Sorensen SE, Greene GW, Eick JD (1981). A critical evaluation of the acute systemic toxicity test for dental alloys using histopathologic criteria. J Biomed Mater Res.

[7] Dammaschke T, Gerth HUV, Zuchner H, Schafer E (2005). Chemical and physical surface and bulk material characterization of white ProRoot MTA and two Portland cements. Dent Mater.

[8] Datta S, Saha DR, Ghosh D, Majumdar T, Bhattacharya S, Mazumder S (2007). Sub-lethal concentration of arsenic interferes with the proliferation of hepatocytes and induces *in vivo* apoptosis in *Clarias batrachus* L. Comp Biochem Physiol C Toxicol Pharmacol.

[9] Demirkaya K, Can Demirdöğen B, Öncel Torun Z, Erdem O, Çetinkaya S, Akay C (2016). *In vivo* evaluation of the effects of hydraulic calcium silicate dental cements on plasma and liver aluminium levels in rats. Eur J Oral Sci.

[10] Demirkaya K, Demirdöğen BC, Torun ZÖ, Erdem O, Çirak E, Tunca YM (2016). Brain aluminium accumulation and oxidative stress in the presence of calcium silicate dental cements. Hum Exp Toxicol.

[11] Duarte MA, Oliveira Demarchi AC, Yamashita JC, Kuga MC, Campos Fraga S (2005). Arsenic release provided by MTA and Portland cement. Oral Surg Oral Med Oral Pathol Oral Radiol Endod.

[12] Garcia LF, Chinelatti MA, Rossetto HL, Pires-de-Souza FC (2014). Solubility and disintegration of new calcium aluminate cement (EndoBinder) containing different radiopacifying agents. J Endod.

[13] Garcia LF, Huck C, Menezes de Oliveira L, Souza PP, Souza Costa CA (2014). Biocompatibility of new calcium aluminate cement: tissue reaction and expression of inflammatory mediators and cytokines. J Endod.

[14] Garcia LF, Huck C, Scardueli CR, Souza Costa CA (2015). Repair of bone defects filled with new calcium aluminate cement (EndoBinder). J Endod.

[15] International Standards Organization (2006). ISO 10993-11:2006: Biological evaluation of medical devices, Part 11. Tests for acute systemic toxicity.

[16] International Standards Organization (2007). ISO 9917-1:2007: Water- based cements - Part 1: Powder/liquid acid-base cements.

[17] International Standards Organization (2008). 7405:2008: Dentistry - Evaluation of biocompatibility of medical devices used in dentistry.

[18] Khalil WA, Eid NF (2013). Biocompatibility of BioAggregate and mineral trioxide aggregate on the liver and kidney. Int Endod J.

[19] Laskin DL (1996). Sinusoidal lining cells and hepatotoxicity. Toxicol Pathol.

[20] Leites AB, Baldissera EZ, Silva AF, Tarquinio S, Botero T, Piva E (2011). Histologic response and tenascin and fibronectin expression after pulp capping in pig primary teeth with mineral trioxide aggregate or calcium hydroxide. Oper Dent.

[21] Leye Benoist F, Gaye Ndiaye F, Kane AW, Benoist HM, Farge P (2012). Evaluation of mineral trioxide aggregate (MTA) versus calcium hydroxide cement (Dycal®) in the formation of a dentine bridge: a randomised controlled trial. Int Dent J.

[22] Min KS, Park HJ, Lee SK, Park SH, Hong CU, Kim HW (2008). Effect of mineral trioxide aggregate on dentin bridge formation and expression of dentin sialoprotein and heme oxygenase-1 in human dental pulp. J Endod.

[23] Minotti PG, Ordinola-Zapata R, Midena RZ, Marciano MA, Cavenago BC, Bramante CM (2015). Rat subcutaneous tissue response to calcium silicate containing different arsenic concentrations. J Appl Oral Sci.

[24] Naghavi N, Ghoddusi J, Sadeghnia HR, Asadpour E, Asgary S (2014). Genotoxicity and cytotoxicity of mineral trioxide aggregate and calcium enriched mixture cements on L929 mouse fibroblast cells. Dent Mater J.

[25] Ozdemir HO, Ozçelik B, Karabucak B, Cehreli ZC (2008). Calcium ion diffusion from mineral trioxide aggregate through simulated root resorption defects. Dent Traumatol.

[26] Piconi C, Maccauro G (1999). Zirconia as a ceramic biomaterial. Biomaterials.

[27] Ramachandran R, Kakar S (2009). Histological patterns in drug-induced liver disease. J Clin Pathol.

[28] Sarkar NK, Caicedo R, Ritwik P, Moiseyeva R, Kawashima I (2005). Physicochemical basis of the biologic properties of mineral trioxide aggregate. J Endod.

[29] Shahi S, Rahimi S, Lotfi M, Yavari H, Gaderian A (2006). A comparative study of the biocompatibility of three root-end filling materials in rat connective tissue. J Endod.

[30] Yaltirik M, Ozbas H, Bilgic B, Issever H (2004). Reactions of connective tissue to mineral trioxide aggregate and amalgam. J Endod.

